# Yacon (*Smallanthus sonchifolius*) root extracts affect laying performance, egg quality, serum biochemical parameters and intestinal microbiota in hens

**DOI:** 10.5713/ab.24.0065

**Published:** 2024-05-07

**Authors:** Zhiwei Wu, Qunli Liu, Zhenting Ruan, Liuchao Wang, Jinghui Fan, Fei Chen, Zhangguo Liu, Lizhi Lu

**Affiliations:** 1 College of Food and Health, College of Animal Science and Technology, Zhejiang Agriculture & Forestry University, Hangzhou 311300, Zhejiang, China; 2 Hospital of Stomatology, Wuhan University, Wuhan 430000, Hubei, China; 3 Institute of Animal Husbandry, Hangzhou Academy of Agricultural Sciences, Hangzhou 310024, Zhejiang, China; 4 Hangzhou Xiaoshan Chicken Breeding Co., Ltd, Hangzhou 311247, Zhejiang, China; 5 Institute of Animal Husbandry and veterinary medicine, Zhejiang Academy of Agricultural Sciences, Hangzhou 310000, Zhejiang, China

**Keywords:** Egg, Hens, Intestinal Microbiota, 16S rRNA, Serum, Yacon

## Abstract

**Objective:**

The objective of this study was to investigate the influence of yacon root extracts (YREs) on productive performance and health of laying hens.

**Methods:**

Six hundred 30-week-old Xiaoshan Chicken layers were divided into 5 groups, control group, antibiotic positive control group, and 3 YREs treatment groups. In a 9-wk feeding experiment, at the end of wk 3, 6, and 9, twenty eggs were collected from each replicate to measure egg qualities. At the end of wk 9, three hen serum samples, and 5 hen cecal content samples were collected from each replicate.

**Results:**

Compared to the control group, 0.8%, 1.6%, and 2.4% YREs treatments could increase hens’ daily feed intake, and YREs supplementation affected daily feed intake in linear manner. YREs did not change egg size, but 0.8% and 2.4% YREs changed egg shape by decreasing the egg shape index and sphericity, and 0.8% YREs tended to improve the eggshell breaking strength. Diet supplemented with 1.6% YREs might decrease yolk color grade but optimize the pH of thick egg white in fresh egg; moreover, 1.6% and 2.4% YREs might be helpful for eggs to inhibit water loss during storage, and YREs supplementation affected water loss rate in linear manner. 2.4% YREs could decrease the serum lactate dehydrogenases (LDH) level, and YREs supplemental levels linearly affected serum LDH content. Finally, YREs could enrich the diversity of intestinal microbiota of hens fed with 0.8% and be beneficial for the relative abundance of phylum Bacteroidota and Halobacterota; 2.4% YREs might increase the abundance of phylum Actinobacteriota and genus *Bifidobacterium*, while decrease genus *Bacteroides*; YREs supplemental levels affected the abundance of phylum Actinobacteriota, and genera *Bifidobacterium* and *Bacteroides* in linear manner.

**Conclusion:**

Dietary supplementation with YREs could affect egg quality, protect the health of organs and exhibit prebiotic activity.

## INTRODUCTION

Yacon (*Smallanthus sonchifolius*), a native plant of South America, is a perennial herbaceous plant of the family Asteraceae. One yacon plant can produce more than 10 kilos of tuberous roots. The yacon root extracts (YREs) exhibit health activities of antioxidant, lipid-lowering [1], promoting the growth of bifidogenic bacteria while inhibiting the establishment of pathogenic and putrefactive bacteria [2], and preventing animal from intestinal inflammation and colorectal cancer [3].

Plant polysaccharides are macromolecules composed of many identical or different monosaccharides linked by glycosidic bonds. They display a variety of biological activities such as immune regulation, antioxidant, antibacterial, anti-tumor, antiviral and hypoglycemic [4]. Yacon root is a potential source of fructooligosaccharides (FOS). Although the yacon root extracts (YREs) contain varieties of components, the major portion of YREs, up to 70%, is FOS [2]. FOS are soluble nondigestible oligosaccharides which can reach the colon intact before undergoing microbial fermentation. FOS supplemented in animal diet could promote the proliferation of beneficial bacteria, inhibit the implantation of pathogenic bacteria, and stimulate immune maturation through short-chain fatty acids produced by intestinal microbiota [2]. Yacon FOS could reduce glycemic index, body weight and the risk of colon cancer [5,6]. Overall, YREs or Yacon FOS might be potential prebiotics and be associated with beneficial effects for consumers.

Eggs are the main output in the laying hen industry, and high-quality eggs are affected by physiological processes in layers which hinges on nutrient utilization and animal health [7]. In laying hen production, prebiotics have been proven to enhance productive efficiency and egg quality [8]. Dietary supplementation of prebiotics has even been applied as a performance-enhancing alternative to in-feed antibiotics [7]. YREs or FOS in YREs might be potential prebiotics to offer benefits to laying hens, however, as far as we know, there is limited information regarding the effects of YREs or YREs FOS on laying hen productive performance and health. Therefore, this study aimed to examine the effect of dietary YREs on laying performance, egg quality, biochemical parameters, and intestinal microbiota in Xiaoshan Chicken layers. Xiaoshan chicken is an indigenous breed originally from Hangzhou, China and is famous for delicious taste. Improving the laying performance of Xiaoshan Chicken layers could bring benefits to local consumers and poultry husbandry.

## MATERIALS AND METHODS

### Animal care

All animal procedures were in strict accordance with the guidelines for the care and use of animals at Zhejiang Agriculture & Forestry University (ZAFUAC202402).

### Animals, diets, and experimental design

Six hundred 30-week-old Xiaoshan Chicken layers were from Hangzhou Xiaoshan Chicken Breeding Co., Ltd (Zhejiang, China). Layers were divided into 5 groups with 6 replicates (20 hens per replicate). Division was performed so that hens in each replicate were very close to the average body weight and laying rate of the flock. Laying hens were raised in 2-tier battery cages with one bird per cage. Feed and water were available *ad libitum*, and the light regime was daily 8 h dark and 16 h light with an intensity of 20 lx. Temperature was between 24°C and 28°C throughout the experiment.

All birds were fed basal diet (non-supplemented ration) for 14 d to adapt to the conditions in the facility, then they were fed the experimental diets for 9 wk. Control group hens continued to be fed with basal diet. Layers in the treatments were fed with the diet supplemented with either 0.8%, 1.6%, or 2.4% YREs. And hens in positive control group were provided with the diet supplemented with 0.4 zinc bacitracin. The basal diet was based on corn and soybean meals and formulated according to the feeding standard of chicken [9]. A full overview of diet composition is presented in Table 1.

YREs (containing 61% FOS, and 77.6% total sugar, 14.8% total phenolics, and 0.69% crude ash) was purchased from Shaanxi Snoot Biotechnology Co., Ltd (Shaanxi, China). Antibiotic Zinc bacitracin (premixed product, containing 10% zinc bacitracin) was purchased from Tianjin Xinxing Pharmaceutical Co., Ltd (Tianjin, China).

### Laying performance and egg quality measurement

#### Laying performance

During the 9 wk-experiment, all eggs laid were collected and weighed daily as an average per replicate. Laying rate was calculated as number of eggs divided by number of hens in the replicate and multiplied 100. Feed intake was measured weekly as average per replicate. Feed efficiency was calculated as g feed intake/g of egg mass. Finally, the egg weight, laying rate, daily feed intake, and feed efficiency were calculated for wk 1 to 3, wk 4 to 6, wk 7 to 9, and wk 1 to 9.

#### Egg qualities

At the end of wk 3, 6 and 9, twenty eggs were collected from each replicate, among which, 10 eggs were immediately measured both eggshell and egg internal quality as fresh eggs, another 10 eggs were measured as preserved eggs after 7 d storage under 16°C temperature and 60% humidity.

After egg weight (m) measurement, the long diameter (L) and wide diameter (W) of eggs were measured using vernier calipers. The geometrical parameters of eggs were calculated as following formulas [10–12]. Egg shape index = (W/L) ×100%, Geometric mean diameter (Dg) = (W×L^2^)^1/3^, Surface area = π×Dg^2^, Volume = (π/6)×L×W^2^, Sphericity = (Dg/L) ×100%.

Eggshell breaking strength was measured by eggshell strength meter (FHK Fujihira Co., Ltd., Tokyo, Japan). Then eggs were individually broken, the thick egg white height (H) and yolk height (Hy) were measured with an albumen height measuring instrument (FHK Fujihira Co., Ltd., Japan). Yolk diameter (Dy) was measured by using vernier calipers. Yolk color was measured with Roche Yolk Color Fan. Thick egg white was stirred first, then pH was measured with acidity meter. Based on these measurements, Haugh unit (HU) was calculated as HU = 100×log(H−1.7×m^0.37^+7.57) [13]; and yolk index (YI) was calculated as YI (%) = (Hy/Dy)×100 [14].

Then the thickness of calcified shell was measured as fol lows [12]. Eggshells were washed with tap water, air-dried, and weighed with an electronic balance. Six pieces of eggshell samples were symmetrically collected at the egg equator, blunt end, and sharp end, the samples were soaked in 5% ethylenediamine tetraacetate (EDTA-Na_2_) solution for 11 minutes, then the outer cuticle and inner shell membrane were removed to obtain the calcified shell. After drying, the thickness of calcified shell samples was individually measured using a spiral micrometer, and the average thickness was calculated.

### Serum biochemical analysis

On the morning of the end of wk 9, three hens were selected from each replicate, and 2 mL venous blood was collected from each hen after fasting for 12 h. The blood was placed at 20°C for 6 to 7 h to separate out serum, and the serum samples were collected and stored at −20°C. Using special kits from Nanjing Jianceng Institute of Biological Engineering (Jiangsu, China), and according to the manufacturers’ procedures, the serum biochemical parameters, such as Albumin (ALB), lactate dehydrogenase (LDH), glutamic oxalacetic transaminase (AST), alkaline phosphatase (AKP), creatinine (CREA), alanine aminotransferase (ALT), triglyceride (TG) and glucose (GLU) were individually measured.

### Intestinal microbiota analysis

Cecal content samples were collected on the morning of the end of wk 9. Five hens were selected from each replicate, and cecal content samples were collected after hens being euthanized by cervical dislocation, the samples were immediately put into liquid nitrogen. Bacterial genome DNA was extracted from each sample using TIAN amp DNA Kit (Tiangen Biotechnology Co., Ltd, Beijing, China). Then the DNA samples were sent to Nuohe Zhiyuan Technology Co., Ltd (Tianjin, China) for sequencing. Primers 515F (5′-GTG CCAGCM GCCGCGGTAA-3′) and 806R (5′-GGACTACH VGGGT WTCTAAT-3′) were used to amplify the hypervariable region V4 of bacterial 16S rRNA gene [15]. The qualified amplicons were purified by magnetic beads, and quantified by enzyme tags, then the amplicons in the same replicate were well mixed in equidensity ratios. The mixed amplicons were used for library construction. After qualify-control for the library, sequencing was performed by Nova Seq 6000. The diversity of intestinal microbiota and bacterial abundances at both phylum and genus levels were analyzed.

### Statistical analysis

The differences of parameters, such as laying performance, egg quality, serum biochemistry, and intestinal microbiota, among 5 groups were analyzed using the software of one-way analysis of variance in SPSS 26.0 (SPSS, Chicago, IL, USA). The means were compared using Duncan’s multiple range test, and each replicate was considered as the experimental unit. Statistical values expressed as means and pooled standard errors of mean. The software of Curvilinear Regression in SPSS 26.0 was used to determine if there were either linear or quadratic relationships between YREs supplemental dosages (0%, 0.8%, 1.6%, and 2.4%) and the evaluated parameters. The threshold of significant difference for all analyses was set as p≤0.05.

## RESULTS

### Effects of YREs on the laying performance

When compared to the control group, during wk 1 to 3, both antibiotic positive control and 0.8% YREs treatments showed significant lower daily feed intake (p<0.01). However, during wk 4 to 6 and wk 7 to 9, treatments with 1.6% and 2.4% YREs, and positive control showed significantly increased daily feed intake (p<0.01). Furthermore, the results of wk 1 to 9 showed that, 3 YREs treatments and positive control group all demonstrated a significant effect on the higher daily feed intake than the control group (p<0.01) (Table 2). Additionally, the results also showed that, there were no significant differences among all 5 groups in laying rate, egg weight and feed efficiency (p>0.05) (Table 2). Briefly, these findings suggested that YREs in hens’ feed could perform as antibiotics to increase the daily feed intake.

Furthermore, the results of wk 4 to 6, wk 7 to 9, and wk 1 to 9 showed that, there were significant both linear and quadratic relationships between the daily feed intake and YREs supplemental levels (p<0.01) (Table 2). This meant that increasing feed intake might linearly depend on YREs supplementation.

### Effects of YREs on the geometrical parameters of eggs

When compared to the control group, eggs collected at wk 3 and wk 6 all showed that egg shape index, sphericity, geometric mean diameter, surface area, and volume among 5 groups were not significantly different (p>0.05) (Table 3). However, eggs collected at wk 9 showed that, 0.8% and 2.4% YREs treatments, and positive control group significantly decreased the egg shape index and sphericity (p<0.05) (Table 3). Above results suggested that, when feeding duration was up to 9 wk, YREs intake tended to change egg shape to be slender and longer, and the effects of YREs supplementation were similar to the antibiotic.

Moreover, there were no significant regression relationships between YREs supplemental levels and examined geometric parameters (p>0.05) (Table 3).

### Effects of YREs on the eggshell quality in fresh eggs

The results of eggs collected at wk 9 showed that 0.8% YREs treatment tended to improve the eggshell breaking strength when compared to the control group (Table 4). However, eggs collected at other evaluation points showed that no significant differences were observed in eggshell percentage (ratio of eggshell weight to intact egg weight), eggshell breaking strength, and calcified shell thickness among 5 groups (p>0.05) (Table 4). These results meant that diet supplemented with YREs for hens had no significant effects on eggshell quality in fresh eggs, except that 0.8% YREs treatment tended to improve the eggshell breaking strength when feeding duration was up to 9 wk.

### Effects of YREs on the interior quality in fresh eggs

At wk 3, no significant differences were observed in yolk color among all groups (p>0.05); but at wk 6, yolk color grades in both 0.8% and 1.6% YREs treatments were significantly lower than the control group (p<0.01) and tended to be less at wk 9 (p>0.05) (Table 5). Similarly, at wk 3, there were no significant differences in pH of thick egg white among all groups (p>0.05), however, compared to the control group, 1.6% YREs treatment significantly decreased the pH at wk 6 (p<0.05) and wk 9 (p<0.01) (Table 5). At any evaluation points, no significant differences were observed in YI and HU among all groups (p>0.05) (Table 5). Briefly, above results suggested that hens fed with 1.6% YREs-supplemented diet might deteriorate yolk color grade but optimize the pH of thick egg white in fresh eggs.

The results also showed that, there were no significant re gression relationships between YREs supplemental levels and tested interior quality in fresh egg (p>0.05) (Table 5).

### Effects of YREs on the interior quality in preserved eggs

At wk 3 and wk 6, no significant differences were observed in water loss rate among 5 groups (p>0.05), but water loss rates of eggs in both 1.6% and 2.4% YREs treatments were significantly less than the control group at wk 9 (p<0.01) (Table 6). Similar to fresh eggs, yolk color in eggs collected at wk 3, showed no significant differences among all groups (p>0.05); however, at wk 6, yolk color grades in both 0.8% and 1.6% YREs treatments were significantly lower than the control group (p<0.01), and at wk 9, yolk color grade in 1.6% YREs treatment tended to be less than the control group (p> 0.05) (Table 6). Eggs collected at wk 6 showed that, after storage, pH of thick egg white in 1.6% and 2.4% YREs treatments was significantly higher than the control group (p< 0.05) (Table 6). Finally, YI and HU among groups were not significantly different (p>0.05) (Table 6). Overall, these results suggested that, during storage, 1.6% and 2.4% YREs treatments might be helpful for eggs to inhibit water loss, but have no positive effects on yolk color grade, and even accelerate the pH of thick egg white increasing faster.

The results also showed significant linear or quadratic re lationships between YREs supplemental levels and water loss rate, yolk color grade, or pH of egg white (p<0.01) (Table 6).

### Effects of YREs on serum biochemical parameters in hens

When compared to the control group, three YREs treatments and positive control group had no significant effects on serum levels of ALT, AST, ALP, ALB, GLU, TG, and CREA in hens (p>0.05); however, supplementation with 2.4% YREs could significantly decrease the serum level of LDH (p<0.05) (Table 7). These results indicated that, when feeding duration was up to 9 weeks, 2.4% YREs intake might decrease serum LDH level.

Furthermore, YREs supplemental levels affected serum level of LDH in both linear and quadratic manners (p<0.05) (Table 7).

### Effects of YREs on the diversity of intestinal microbiota in hens

We evaluated effects of YREs intake on gut microbial community using α-diversity indices. No significant differences were observed in some indices, such as Shannon index, Simpson index and Goods coverage among all 5 groups (p>0.05) (Table 8). However, other diversity indices, such as the observed species, Chao1, abundance-based coverage estimator (ACE), and phylogenetic diversity (PD) whole tree, were significantly improved by 0.8% YREs group when compared to the control group (p<0.05). This suggested that hens fed with 0.8% YREs could enrich the diversity of intestinal microbiota.

Furthermore, increases in YREs supplemental levels had a quadratic effect on PD whole tree (p<0.05) (Table 8).

### Effects of YREs on the relative abundances of top 10 intestinal bacteria at phylum level

The relative abundance of phyla Firmicutes, Fusobacteriota, Proteobacteria, Verrucomicrobiota, Campylobacterota, unidentified-Bacteria, and Euryarchaeota showed no significant changes among all 5 groups (p>0.05) (Table 9). However, when compared to the control group, 0.8% YREs treatment significantly increased the relative abundance of both phyla Bacteroidota and Halobacterota (p<0.05); and 2.4% YREs significantly increased the relative abundance of phylum Actinobacteriota (p<0.05). Briefly, these findings suggested that hens feeding diet with 0.8% or 2.4% YREs was beneficial for the relative abundance of phylum Bacteroidota, Halobacterota, or Actinobacteriota.

The abundance of Actinobacteriota increased with YREs supplemental levels in both linear and quadratic manners (p<0.05) (Table 9).

### Effects of YREs on the relative abundances of top 10 intestinal bacteria at genus level

At the genus level, no significant differences were observed in the relative abundance of genera *Lactobacillus*, *Fusobacterium*, *Megamonas*, *Enterococcus*, *Romboutsia*, *Fournierella*, *Rikenellacea-RC9-gut-group*, and *Clostridium-sensu-stricto-1* among all groups (p>0.05) (Table 10). However, compared to the control group, 2.4% YREs-received hens showed significantly increase in the abundance of *Bifidobacterium* spp. while decrease in *Bacteroides* spp. (p<0.05).

Furthermore, increase in YREs supplementation had both linear and quadratic effects on the abundance of genera *Bifidobacterium* and *Bacteroides* (p<0.05) (Table 10).

## DISCUSSION

### Effects of dietary YREs on egg qualities

Egg profile is not only useful for research on ecological morphology [16], but also meaningful for the poultry industry to predict eggshell quality, breeding egg hatchability [11], and eggshell behavior under mechanical loading or thermal treatments during food processing [17]. Present results showed that YREs supplemented for layers had no effects on egg size parameters (geometric mean diameter, surface area, and volume), but decreased the egg shape parameters (egg shape index, and sphericity), eggs tended to be relatively slender and longer. Previous study also showed that red yeast (a source of mannan-oligosaccharides) supplementation had no significant effect on egg surface area but improved the egg shape index [7]. It was reported that there was correlation between shape index and eggshell breaking strength, indicating the rounder chicken eggs being somewhat more resistant to breakage than more elongated eggs [18]. However, quail eggs with a shape index >78% had a higher hatchability rate than that of rounder eggs [19].

Our results showed that YREs supplementation had no significant effects on calcified shell thickness and eggshell percentage, but 0.8% YREs tended to improve the eggshell breaking strength when feeding duration was up to 9 weeks. Previous study reported that eggshell breaking strength was increased by supplementation with xylo-oligosaccharides and wheat bran [20]. Cracked and damaged eggs cause substantial economic loss to the egg and breeding industry. It is suggested that the increased eggshell breaking strength might related to calcium metabolism in laying hens [21]; on the other hand, it is also suggested that eggshell strength might be affected partially by ultrastructure of the eggshell, and steady and continuous absorption of calcium might be beneficial for its ultrastructure [22]. It has been reported that inulin-type fructan could enhance calcium absorption and retention in animal femurs [23]. However, further investigation is required to determine whether YREs has the direct effects on calcium absorption/metabolism or on the ultrastructure of eggshells.

Present results showed that 1.6% YREs supplementation for hens decreased the yolk color grade but optimized the pH of thick egg white in fresh eggs. It is reported that yolk color grade was reduced by feeding xylo-oligosaccharides or wheat bran [20]. Yolk colour is an important quality trait of eggs, because consumers have a greater preference for redness of yolks and for precursors of retinol or vitamin A. During yolk formation, considerable amounts of various carotenoids and pigment matters deposit into the avian oocyte. The carotenoids play an important role in the egg yolk coloration. It is suggested that changes of yolk color are largely associated with the ingredients used in diets [24]. In our current study, the yolk discoloration might result from YREs attenuating the absorption or deposition of dietary carotenoids. The effects of indigestible oligosaccharides on pH of thick egg white has not been previously reported. The optimized egg white pH in this study could benefit from the short chain fatty acids (SCFA) produced by oligosaccharides fermentation which might decrease intestinal and even body fluid pH.

There is rather limited evidence regarding the effects of oligosaccharides on qualities in preserved eggs. Current results showed that 1.6% or 2.4% YREs treatment could be helpful inhibiting water loss from eggs during storage. The lost water mainly originated from egg white during the short-term storage, therefore, it is possible dietary YREs improves the water holding capability of egg white by changing fine structures of some albumen proteins or improves the ultrastructure of eggshell to inhibit water loss. On the other hand, present study showed that 0.8% or 1.6% YREs intake induced a lower yolk color grade, which might be attributed to deteriorated yolk color grade in fresh eggs, therefore, it implied that YREs intake has no positive effects on yolk color during storage. Finally, present study showed that pH of thick egg white in eggs from 1.6% or 2.4% YREs group was prone to rise faster, this means that YREs intake is not beneficial for maintaining albumen pH during storage. The faster increase of albumen pH during storage usually results from a lower buffering capacity of egg white [25], whereas the lower albumen buffering capacity is related to the easier CO_2_ loss from albumen [26]. Therefore, a faster increase of albumen pH might relate to a more rapid release of CO_2_ from albumen or through a higher permeability of the eggshell [27]. However, our findings showed that YREs intake might improve eggshell strength, which is helpful in inhibiting water loss during egg storage. Therefore, the current YREs induced more rapid increase of albumen pH might be caused by a change in physicochemical properties of albumen proteins rather than by weaker shell quality.

### Effects of YREs on serum biochemical parameters of hens

In some forms of hepatic and cardiac toxicity, the levels of several cytosolic, mitochondrial, and membrane-associated enzymes are higher in the plasma, because the lesions in the cell membrane facilitate the entry of intracellular enzymes into the systemic circulation. Lactate dehydrogenases is a glycolytic enzyme commonly found in mammals. The serum level of LDH always increases when the pathological process affects the integrity of cells, especially hepatic and cardiac cells [28]. LDH in the serum are usually measured as biomarkers of tissue damage. It was reported that FOS intake could reduce mouse serum LDH level which might be due to reduced liver and muscle damage [29]. Our results showed that 2.4% YREs supplementation for hens could significantly decrease the serum LDH level and increases in YREs supplementation had both linear and quadratic effects on serum LDH level. These results indicate the hepatoprotective potential when some dosage of YREs is added for hens, probably because it maintains plasmatic membrane integrity and recovers hepatic tissue.

### Effects of dietary YREs on intestinal microbiota in hens

YREs contain abundant indigestible FOS that can reach avian caeca or mammal colons where microbial fermentation can produce SCFA. Consequently, fermentation of indigestible carbohydrates affects the gut microbial community. It is reported that mice receiving a 10% yacon-containing diet had an increase in α-diversity indices of intestinal microbiota, such as operational taxonomic units (OTU) richness estimating parameters, observed species and Chao 1 index [3]. Our results showed that 0.8% YREs treatment increased diversity parameters in intestinal microbiota, such as the observed species, Chao1, ACE, and PD whole tree. This indicates that 0.8% YREs added to layers’ feed might improve the OTU richness of intestinal microbiota.

SCFA produced by fermentation of FOS can favor the growth of bifidobacteria in the gut, while inhibiting pathogenic populations [30]. It was reported that 10% yacon intake led to modulating mouse intestinal microbial composition, such as higher abundances of phyla Actinobacteria and Firmicutes, and lower abundances of phylum Bacteroidetes; at the same time, yacon powder intake increased abundance of genera *Bifidobacterium*, *Lactobacillus*, and *Allobaculum*, and inhibited genera *Bacteroides*, *Desulfovibrio*, and *Akkermansia* [3]. In agreement with these results, our study showed that 2.4% YREs supplementation for hens tended to increase relative abundance of phylum Actinobacteriota and genus *Bifidobacterium*, while decreased genus *Bacteroides*. However, 0.8% YREs group tended to increase the abundance of phylum Bacteroidota and genus *Bacteroides*. Actinobacteriota plays an important role in the decomposition of organic matter [31]. *Bifidobacterium* spp. belongs to phylum Actinobacteriota and can produce SCFA as metabolites from several substrates such as glycerol and glucose, finally, SCFA favors the growth of commensal bacteria instead of pathogenic bacteria [32]. Furthermore, *Bifidobacterium* is a bacterial community with importance in reducing blood lipid and improving immunity and antioxidant activity [33]. Phylum Bacteroidota plays an important role in maintaining the balance of gut microbiota and improving intestinal metabolism [34]. Genomes of Bacteroidaceae are enriched for genes involved in degradation of complex polysaccharides and their metabolism produces acetate, propionate, or succinate [35]. Genus *Bacteroides* may acidify nutrient broths *in vitro* nearly as effectively as *Lactobacilli* spp., so the fermentation and production of organic acid is quite extensive [36]. Therefore, 2.4% YREs supplementation for hens might exhibit prebiotic activity through promoting growth of *Bifidobacterium* spp.; the decreasing abundance of genus *Bacteroides* possibly affected by normalization to increase the relative abundance of genus *Bifidobacterium*. On the other hand, it is possible that 0.8% YREs intake elicits a bifidogenic effect by selectively stimulating the proliferation of phyla Bacteroidota and genus *Bacteroides*.

## CONCLUSION

Present study demonstrated that YREs supplementation in hens’ diet could improve the eggshell breaking strength; optimize the pH of thick egg white in fresh eggs and inhibit water loss from eggs during storage. Our findings also presented that dietary YREs could decrease the serum LDH level and enrich some beneficial bacteria in the intestine. Overall, YREs supplementation in hen diet could affect egg quality characteristics, protect the health of organs, and exhibit prebiotic activity.

## Figures and Tables

**Table 1:** Basal diet composition and nutrient composition

Items	Content
Ingredients (%)	
Corn	60
Soybean meal	28
Calcium dihydrogen phosphate	2
Limestone powder	5
Premix^1)^	5
Combination	100
Nutrients^2)^	
Metabolic energy (MJ/kg)	10.33
Crude protein (%)	16.70
Total phosphorus (%)	0.65
Effective phosphorus (%)	0.35
Lysine (%)	0.77
Methionine (%)	0.48

1) per kilogram of premix contains: vitamin A 8,000 IU, vitamin B_2_ 4.5 mg, vitamin B_5_ 2 mg, vitamin B_6_ 0.2 mg, vitamin B_12_ 0.02 mg, vitamin D_3_ 2,800 IU, vitamin E 23 IU, vitamin K_3_ 2.6 mg, biotin 0.0075 mg, folic acid 0.05 mg, calcium pantothenate 15 mg, niacin 30.5 mg, sodium chloride 37.5 mg, copper (CuSO_4_·5H_2_O) 17.5 mg, ferrum (FeSO_4_·7H_2_O) 60 mg, manganese (MnSO_4_·H_2_O) 65 mg, zinc (ZnO) 70 mg, iodine (KI) 0.75 mg, selenium (Na_2_SeO_3_) 0.2 mg.

2) Nutrients were calculated based on dry matter.

**Table 2:** Effects of dietary YREs on laying performance of hens

Parameters	Trial period	Treatment groups							Linear regression		Quadratic regression	
		
		Control	0.4‰ ZB^1)^	0.8% YREs	1.6% YREs	2.4% YREs	SEM	p-value	R^2^	p-value	R^2^	p-value
Feed intake (g/d)	wk 1 to 3	113.72^a^	112.33^bc^	112.12^c^	113.32^ab^	113.80^a^	0.17	<0.01	0.03	0.40	0.37	<0.01
	Wk 4 to 6	100.82^d^	109.42^b^	110.46^ab^	106.39^c^	111.5^a^	0.74	<0.01	0.53	<0.01	0.59	<0.01
	Wk 7 to 9	105.01^b^	106.32^a^	105.31^ab^	106.37^a^	106.32^a^	0.17	0.01	0.36	<0.01	0.37	<0.01
	Wk 1 to 9	106.52^c^	109.36^b^	109.30^b^	108.69^b^	110.57^a^	0.28	<0.01	0.63	<0.01	0.65	<0.01
Mortality rate (%)	Wk 1 to 9	0.00	0.00	0.00	0.00	0.00	0.00	1.00	0.00	1.00	0.00	1.00
Laying rate (%)	wk 1 to 3	66.93	67.95	69.21	70.80	71.03	1.05	0.71	0.08	0.17	0.09	0.36
	Wk 4 to 6	67.06	64.55	65.67	63.09	66.69	1.23	0.86	<0.01	0.76	0.05	0.61
	Wk 7 to 9	67.06	63.49	63.06	65.47	63.74	1.21	0.69	0.05	0.30	0.05	0.59
	Wk 1 to 9	67.02	65.33	65.98	66.45	66.49	0.98	0.99	<0.01	0.91	<0.01	0.97
Egg weight (g)	wk 1 to 3	47.07	46.83	47.24	47.71	47.97	0.25	0.61	0.07	0.22	0.07	0.48
	Wk 4 to 6	48.08	47.31	47.69	47.72	48.36	0.24	0.73	<0.01	0.69	0.05	0.52
	Wk 7 to 9	48.16	47.80	47.91	48.47	48.61	0.19	0.65	0.04	0.35	0.05	0.59
	Wk 1 to 9	47.77	47.67	47.61	48.48	48.31	0.24	0.72	0.05	0.28	0.05	0.57
Ratio of feed to egg	wk 1 to 3	2.86	3.26	2.90	3.07	3.34	0.07	0.14	0.21	0.03	0.23	0.07
	Wk 4 to 6	3.40	3.72	3.95	3.23	3.45	0.17	0.70	0.01	0.76	<0.01	0.89
	Wk 7 to 9	3.25	3.52	3.55	3.39	3.70	0.08	0.44	0.09	0.16	0.09	0.38
	wk 1 to 9	3.17	3.57	3.47	3.23	3.50	0.09	0.53	0.03	0.42	0.03	0.73

YREs, yacon root extracts; SEM, standard error of mean.

1) 0.4‰ ZB represents 0.4‰ zinc bacitracin.

a–d Means in a row with different superscripts differ (p<0.05).

**Table 3:** Effects of dietary YREs on egg geometric parameters

Parameters	Trial period	Treatment groups							Linear regression		Quadratic regression	
		
		Control	0.4‰ ZB^1)^	0.8% YREs	1.6% YREs	2.4% YREs	SEM	p-value	R^2^	p-value	R^2^	p-value
Egg shape index (%)	wk 3	74.78	73.36	73.16	73.80	73.94	0.43	0.84	<0.01	0.77	0.02	0.67
	wk 6	73.67	73.24	72.36	73.82	73.27	0.27	0.58	<0.01	0.69	0.03	0.26
	wk 9	74.25^a^	72.02^c^	72.67^bc^	74.07^ab^	73.07^bc^	0.26	0.02	<0.01	0.30	0.01	0.48
Sphericity (%)	wk 3	90.75	90.17	90.08	90.35	90.41	0.18	0.83	<0.01	0.95	0.07	0.22
	wk 6	90.30	89.90	89.95	90.41	90.13	0.12	0.82	<0.01	0.55	<0.01	0.71
	wk 9	90.53^a^	89.62^c^	89.88^bc^	90.46^a^	90.05^b^	0.11	0.02	<0.01	0.95	0.01	0.53
Geometric mean diameter (mm)	wk 3	47.81	48.18	48.64	48.64	47.97	0.18	0.49	<0.01	0.78	0.02	0.67
	wk 6	48.75	48.58	48.73	48.71	49.00	0.13	0.89	<0.01	0.63	0.01	0.50
	wk 9	48.33	48.57	49.01	48.35	48.46	0.13	0.46	<0.01	0.30	0.01	0.47
Surface area (mm^2^)	wk 3	7,185.54	7,295.86	7,442.18	7,437.41	7,237.39	54.51	0.48	<0.01	0.94	0.07	0.22
	wk 6	7,472.36	7,365.32	7,466.46	7,461.66	7,551.03	36.85	0.65	<0.01	0.53	<0.01	0.70
	wk 9	7,348.02	7,422.77	7,559.79	7,353.03	7,382.19	40.39	0.43	<0.01	0.92	0.01	0.50
Volume (mm^3^)	wk 3	42,794.85	42,964.29	44,038.85	44,516.82	42,781.92	382.04	0.47	<0.01	0.97	0.06	0.26
	wk 6	44,705.65	44,281.61	44,167.69	44,151.04	45,201.62	302.02	0.80	<0.01	0.65	0.02	0.44
	wk 9	43,969.41	43,326.87	44,652.56	43,947.65	43,566.26	313.16	0.75	<0.01	0.57	<0.01	0.60

YREs, yacon root extracts; SEM, standard error of mean.

1) 0.4‰ ZB represents 0.4‰ zinc bacitracin.

a–c Means in a row with different superscripts differ (p<0.05).

**Table 4:** Effects of dietary YREs on eggshell quality

Parameters	Trial period	Treatment groups							Linear regression		Quadratic regression	
		
		Control	0.4‰ ZB^1)^	0.8% YREs	1.6% YREs	2.4% YREs	SEM	p-value	R^2^	p-value	R^2^	p-value
Eggshell percentage (%)	wk 3	9.24	9.06	8.87	9.02	9.02	0.10	0.87	<0.01	0.69	0.02	0.67
	wk 6	9.12	8.95	9.03	8.84	9.19	0.07	0.52	<0.01	0.98	0.02	0.37
	wk 9	8.85	8.90	8.92	8.68	8.86	0.06	0.81	<0.01	0.31	<0.01	0.56
Eggshell strength (kgf)	wk 3	3.57	3.90	3.66	4.07	3.78	0.09	0.43	0.03	0.28	0.05	0.36
	wk 6	3.69	3.76	3.56	3.56	3.86	0.05	0.26	0.01	0.31	0.05	0.08
	wk 9	3.64^ab^	3.65^ab^	3.92^a^	3.49^b^	3.37^b^	0.06	0.04	0.03	0.05	0.05	0.04
Calcified shell thickness (mm)	wk 3	0.30	0.29	0.29	0.29	0.29	<0.01	0.96	<0.01	0.81	0.02	0.73
	wk 6	0.30	0.16	0.28	0.22	0.22	0.02	0.26	0.04	0.06	0.04	0.16
	wk 9	0.29	0.29	0.30	0.29	0.29	<0.01	0.16	<0.01	0.32	0.02	0.28

YREs, yacon root extracts; SEM, standard error of mean.

1) 0.4‰ ZB represents 0.4‰ zinc bacitracin.

a,b Means in a row with different superscripts differ (p<0.05).

**Table 5:** Effects of dietary YREs on interior quality in fresh eggs

Parameters	Trial period	Treatment groups							Linear regression		Quadratic regression	
		
		Control	0.4‰ ZB^1)^	0.8% YREs	1.6% YREs	2.4% YREs	SEM	p-value	R^2^	p-value	R^2^	p-value
Yolk color	wk 3	8.44	7.81	7.66	8.50	8.08	0.16	0.36	<0.01	0.98	<0.01	0.92
	wk 6	7.33^a^	7.31^a^	6.00^b^	6.08^b^	6.72^ab^	0.12	<0.01	0.03	0.11	0.15	<0.01
	wk 9	6.75	6.38	6.29	6.28	6.72	0.08	0.15	<0.01	0.74	0.05	0.04
Yolk index (%)	wk 3	46.47	45.57	46.59	46.56	46.07	0.29	0.78	<0.01	0.63	0.01	0.79
	wk 6	44.12	43.61	43.15	43.42	43.76	0.18	0.49	<0.01	0.56	0.03	0.16
	wk 9	43.40	43.87	44.05	44.51	43.92	0.23	0.62	<0.01	0.31	0.02	0.30
Haugh unit	wk 3	83.46	82.55	86.51	84.34	82.63	1.04	0.74	<0.01	0.59	0.03	0.54
	wk 6	82.23	79.47	81.20	80.30	81.53	0.64	0.67	<0.01	0.62	<0.01	0.66
	wk 9	79.80	78.81	81.00	83.70	79.96	0.68	0.24	<0.01	0.57	0.02	0.27
pH of thick egg white	wk 3	8.15	8.27	8.24	8.27	8.24	0.04	0.91	<0.01	0.53	0.02	0.67
	wk 6	8.37^a^	8.43^a^	8.45^a^	8.21^b^	8.29^ab^	0.03	0.04	<0.01	0.46	<0.01	0.62
	wk 9	8.38^a^	8.49^a^	8.41^a^	8.17^b^	8.36^a^	0.02	<0.01	0.02	0.10	0.04	0.08

YREs, yacon root extracts; SEM, standard error of mean.

1) 0.4‰ ZB represents 0.4‰ zinc bacitracin.

a,b Means in a row with different superscripts differ (p<0.05).

**Table 6:** Effects of dietary YREs on interior quality in preserved eggs

Parameters	Trial period	Treatment groups							Linear regression		Quadratic regression	
		
		Control	0.4‰ ZB^1)^	0.8% YREs	1.6% YREs	2.4% YREs	SEM	p-value	R^2^	p-value	R^2^	p-value
Water loss rate (%)	wk 3	0.86	0.93	0.95	0.86	0.97	0.03	0.48	0.02	0.33	0.02	0.63
	wk 6	1.24	1.11	1.20	1.20	1.21	0.02	0.35	<0.01	0.65	<0.01	0.73
	wk 9	1.25^b^	1.33^a^	1.24^b^	1.08^c^	1.10^c^	0.02	<0.01	0.07	<0.01	0.07	0.02
Yolk color	wk 3	7.72	6.72	6.41	6.66	7.16	0.16	0.08	0.01	0.46	0.13	0.04
	wk 6	5.99^a^	6.08^a^	5.14^b^	5.25^b^	6.06^a^	0.10	<0.01	<0.01	0.63	0.11	<0.01
	wk 9	6.00	5.74	5.89	5.40	5.92	0.08	0.09	<0.01	0.31	0.03	0.13
Yolk index (%)	wk 3	41.89	42.39	43.19	42.56	43.21	0.31	0.63	0.03	0.26	0.04	0.46
	wk 6	39.60	39.94	40.60	39.71	39.10	0.18	0.11	0.02	0.14	0.06	0.03
	wk 9	40.52	41.19	41.10	41.13	40.91	0.18	0.73	<0.01	0.45	0.01	0.46
Haugh unit	wk 3	67.79	67.31	69.70	68.21	70.96	0.96	0.75	0.02	0.41	0.02	0.70
	wk 6	67.57	66.66	69.94	65.41	64.35	0.67	0.08	0.04	0.04	0.05	0.06
	wk 9	65.83	64.08	67.44	67.21	65.43	0.71	0.59	<0.01	0.91	<0.01	0.59
pH of thick egg white	wk 3	9.49	9.55	9.57	9.59	9.57	0.01	0.28	0.08	0.06	0.13	0.05
	wk 6	9.28^c^	9.32^ab^	9.29^c^	9.33^ab^	9.36^a^	0.01	0.02	0.12	<0.01	0.12	<0.01
	wk 9	9.31	9.31	9.31	9.28	9.29	0.01	0.86	<0.01	0.48	<0.01	0.76

YREs, yacon root extracts; SEM, standard error of mean.

1) 0.4‰ ZB represents 0.4‰ zinc bacitracin.

a–c Means in a row with different superscripts differ (p<0.05).

**Table 7:** Effects of dietary YREs on serum biochemical parameters in layers

Parameters	Treatment groups							Linear regression		Quadratic regression	
		
	Control	0.4‰ ZB^1)^	0.8% YREs	1.6% YREs	2.4% YREs	SEM	p-value	R^2^	p-value	R^2^	p-value
ALT (U/L)	1.93	4.79	2.02	3.26	2.28	0.46	0.22	0.02	0.56	0.03	0.73
AST (U/L)	18.76	23.59	27.49	23.17	20.77	1.40	0.39	<0.01	0.99	0.13	0.25
ALP (U/L)	156.34	148.29	198.94	197.72	136.39	10.58	0.18	0.02	0.52	0.22	0.08
LDH (U/L)	534.79^a^	489.01^ab^	523.73^a^	524.90^a^	441.63^b^	11.21	0.03	0.26	0.01	0.35	0.01
ALB (g/L)	26.04	26.15	25.82	27.66	27.17	0.76	0.94	0.02	0.50	0.02	0.80
GLU (mmol/L)	17.04	19.30	17.94	16.28	19.68	0.74	0.59	0.03	0.44	0.06	0.57
TG (mmol/L)	9.76	9.86	11.01	8.77	8.65	0.53	0.65	0.05	0.30	0.06	0.51
CREA (μmol/L)	102.51	123.50	98.44	125.50	111.23	9.24	0.87	0.01	0.61	0.01	0.86

YREs, yacon root extracts; SEM, standard error of mean; ALT, alanine aminotransferase; AST, aspartate aminotransferase; ALP, alkaline phosphatase; LDH, lactate dehydrogenase; ALB, albumin; GLU, serum glucose; TG, triglyceride; CREA, creatinine.

1) 0.4‰ ZB represents 0.4‰ zinc bacitracin.

a,b Means in a row with different superscripts differ (p<0.05).

**Table 8:** Effects of dietary YREs on the diversity of intestinal microbiota in layers

Parameters	Treatment groups							Linear regression		Quadratic regression	
		
	Control	0.4‰ ZB^1)^	0.8% YREs	1.6% YREs	2.4% YREs	SEM	p-value	R^2^	p-value	R^2^	p-value
Observed species	684.66^bc^	814.16^ab^	845.66^a^	674.50^c^	680.16^c^	23.10	0.02	0.03	0.42	0.14	0.22
Shannon	4.90	5.42	6.10	4.60	5.08	0.21	0.18	0.01	0.65	0.04	0.67
Simpson	0.85	0.87	0.92	0.81	0.88	0.01	0.26	<0.01	0.97	<0.01	0.10
Chao1	744.74^bc^	874.51^ab^	914.17^a^	730.07^c^	730.23^c^	24.31	0.02	0.04	0.36	0.15	0.19
ACE	747.00^bc^	881.93^ab^	923.88^a^	740.85^c^	738.00^c^	24.55	0.02	0.03	0.40	0.15	0.18
Goods coverage	0.9980	0.9980	0.9976	0.9980	0.9979	<0.01	0.09	0.07	0.21	0.20	0.10
PD whole tree	60.44^b^	70.69^ab^	88.12^a^	78.20^ab^	63.273^b^	3.17	0.02	<0.01	0.97	0.34	0.01

YREs, yacon root extracts; SEM, standard error of mean; ACE, abundance-based coverage estimator; PD, phylogenetic diversity.

1) 0.4‰ ZB represents 0.4‰ zinc bacitracin.

a–c Means in a row with different superscripts differ (p<0.05).

**Table 9:** Effects of dietary YREs on relative abundances of top 10 intestinal bacteria at phylum level

Parameters	Treatment groups							Linear regression		Quadratic regression	
		
	Control	0.4‰ ZB^1)^	0.8% YREs	1.6% YREs	2.4% YREs	SEM	p-value	R^2^	p-value	R^2^	p-value
*Firmicutes* (%)	59.31	44.04	37.64	52.15	49.12	3.22	0.28	0.01	0.63	0.08	0.41
*Fusobacteriota* (%)	16.76	19.52	18.59	25.41	22.10	2.26	0.80	0.05	0.32	0.06	0.54
*Bacteroidota* (%)	18.52^b^	22.86^ab^	33.28^a^	16.14^b^	10.62^b^	2.48	0.04	0.11	0.12	0.24	0.05
*Actinobacteriota* (%)	0.59^b^	3.39^b^	0.70^b^	0.84^b^	12.89^a^	1.42	0.01	0.27	<0.01	0.41	<0.01
*Proteobacteria* (%)	1.17	2.62	1.84	1.62	1.85	0.22	0.34	0.07	0.22	0.09	0.38
*Verrucomicrobiota* (%)	0.14	0.26	1.23	0.06	0.24	0.20	0.34	<0.01	0.70	0.04	0.63
*Halobacterota* (%)	0.09^c^	2.39^a^	0.79^b^	0.12^c^	<0.01^c^	0.24	<0.01	0.03	0.41	0.16	0.16
*Campylobacterota* (%)	0.60	0.15	0.49	0.35	0.93	0.13	0.43	0.02	0.57	0.07	0.49
*Unidentified-Bacteria* (%)	0.86	1.43	1.40	0.66	0.77	0.12	0.09	0.05	0.32	0.09	0.37
*Euryarchaeota* (%)	<0.01	<0.01	0.64	0.05	0.02	0.11	0.23	0.01	0.65	0.08	0.42

YREs, yacon root extracts; SEM, standard error of mean.

1) 0.4‰ ZB represents 0.4‰ zinc bacitracin.

a–c Means in a row with different superscripts differ (p<0.05).

**Table 10:** Effects of dietary YREs on relative abundances of top 10 intestinal bacteria at genus level

Parameters	Treatment groups							Linear regression		Quadratic regression	
		
	Control	0.4‰ ZB^1)^	0.8% YREs	1.6% YREs	2.4% YREs	SEM	p-value	R^2^	p-value	R^2^	p-value
*Lactobacillus* (%)	38.50	22.07	14.15	36.53	28.05	3.61	0.17	<0.01	0.81	0.04	0.62
*Fusobacterium* (%)	16.76	19.52	18.59	25.41	22.10	2.26	0.80	0.05	0.32	0.06	0.54
*Megamonas* (%)	0.89	2.26	1.45	1.81	0.40	0.33	0.43	<0.01	0.71	0.11	0.30
*Enterococcus* (%)	5.23	0.04	0.15	0.09	0.20	0.89	0.27	0.10	0.13	0.16	0.16
*Bifidobacterium* (%)	<0.01^b^	2.79^b^	0.12^b^	0.33^b^	11.25^a^	1.29	0.01	0.27	<0.01	0.41	<0.01
*Bacteroides* (%)	11.67^ab^	10.59^abc^	13.42^a^	5.53^bc^	4.20^c^	1.16	0.03	0.27	<0.01	0.29	0.03
*Romboutsia* (%)	2.96	6.89	5.79	2.61	3.35	0.77	0.31	<0.01	0.73	0.04	0.68
*Fournierella* (%)	1.12	0.64	1.42	1.43	2.10	0.18	0.13	0.11	0.11	0.12	0.26
*Rikenellaceae-RC9-gut-group* (%)	2.29	3.87	6.19	3.80	2.71	0.59	0.26	<0.01	0.86	0.14	0.21
*Clostridium-sensu-stricto-1* (%)	0.34	0.98	1.27	0.54	0.26	0.17	0.27	0.02	0.52	0.17	0.15

YREs, yacon root extracts; SEM, standard error of mean.

1) 0.4‰ ZB represents 0.4‰ zinc bacitracin.

a–c Means in a row with different superscripts differ (p<0.05).
